# Draft Genome Sequence of Propionibacterium acnes subsp. elongatum Strain Asn12

**DOI:** 10.1128/MRA.00801-18

**Published:** 2018-07-19

**Authors:** Andrew McDowell, Judit Hunyadkürti, Márta Magyari, Andrea Vörös, Balázs Horváth, Sheila Patrick, István Nagy

**Affiliations:** a Northern Ireland Centre for Stratified Medicine, School of Biomedical Sciences, Ulster University, Londonderry, United Kingdom; b Centre for Experimental Medicine, School of Medicine, Dentistry & Biomedical Sciences, Queen’s University, Belfast, United Kingdom; c Institute of Biochemistry, Biological Research Centre of the Hungarian Academy of Sciences, Szeged, Hungary; d SeqOmics Biotechnology Ltd., Mórahalom, Hungary; e ATGandCo Biotechnology Ltd., Mórahalom, Hungary; Louisiana State University

## Abstract

Propionibacterium acnes, a non-spore-forming anaerobic Gram-positive bacterium, has been linked to a wide range of opportunistic human infections and conditions, most notably acne vulgaris. Here, we present the draft genome sequence of P. acnes subsp. elongatum strain Asn12, isolated from spinal disc tissue (in the United Kingdom).

## Abstract (precis)

Propionibacterium acnes, a non-spore-forming anaerobic Gram-positive bacterium, has been linked to a wide range of opportunistic human infections and conditions, most notably acne vulgaris. Here, we present the draft genome sequence of P. acnes subsp.

## ANNOUNCEMENT

The Gram-positive anaerobic bacterium Propionibacterium acnes forms part of the normal microbiota on human skin and mucosal surfaces. While normally associated with skin health, P. acnes is also an opportunistic pathogen linked with a range of human infections and clinical conditions, such as acne vulgaris (1), prosthetic joint infection (2), prostate cancer (3), sarcoidosis (4), progressive macular hypomelanosis (PMH) (5), and degenerative disc disease (6). Ever since we showed that distinct strains of P. acnes induce different gene expression patterns in human keratinocytes (7) and sebocytes (8), further advances in our understanding of the intraspecies phylogeny of P. acnes have occurred. Distinct phylogroups have been discovered and specific strains or sequence types (STs) associated with human health or disease revealed (9). In-depth studies of the phylogenetic and taxonomic heterogeneity of P. acnes have ultimately led to the recent proposal of the type I, II, and III phylogroups as distinct subspecies known as P. acnes subsp. acnes, P. acnes subsp. defendens, and P. acnes subsp. elongatum, respectively.

Here, we present the draft genome sequence of P. acnes subsp. elongatum strain Asn12 (10) that was isolated from spinal disc tissue (in the United Kingdom). The culture conditions and genomic DNA isolation methods were as published previously (11, 12). Sequencing libraries with ∼500-bp inserts were prepared from 500 ng of input DNA using the NEBNext DNA library prep master mix for Illumina. Genome sequencing was performed on an Illumina MiSeq instrument, which generated 1,380,920 2 × 250-bp reads and yielded ∼95-fold coverage. Assembly was performed using the Genomics Workbench 11.0 (Qiagen). Gap closing was accomplished using PCR (primers are available on request), followed by Sanger sequencing, as described previously (13). Automatic annotation of the genome was performed using the NCBI Prokaryotic Genomes Annotation Pipeline (PGAP) version 4.5 (https://www.ncbi.nlm.nih.gov/genomes/static/Pipeline.html). We have assembled the genome of P. acnes subsp. elongatum strain Asn12 into 2 contigs, with 2,484,878 bp, 2,422 putative coding sequences, 45 tRNAs, and 9 rRNAs.

To date, the majority of the P. acnes genomes sequenced belong to P. acnes subsp. acnes, with only four genomes of P. acnes subsp. elongatum strains (HL201PA1, JCM18909, PMH5, and PMH7) available (14, 15). This is not surprising since this subspecies has rarely been cultured from healthy facial skin, which is a primary skin sampling site, or from opportunistic infections or acne patients; strains from this subspecies, however, have been recently linked with the skin condition PMH (5, 16). Furthermore, type III strains are more frequently found on the back and abdomen than on other body sites, suggesting that this may be their preferred niche (16). P. acnes subsp. elongatum strain Asn12 belongs to ribotype 9 and, on the basis of our eight-gene multilocus sequence typing (MLST_8_) scheme (17), belongs to the ST33 lineage and clonal complex 77. As isolates PMH5 and PMH7 also belong to ST33, it will be of particular importance to sequence P. acnes subsp. elongatum isolates from other STs.

### Data availability.

This whole-genome shotgun project has been deposited at DDBJ/EMBL/GenBank under the accession number QKRC00000000. The version described in this paper is version QKRC01000000.
